# Tranexamic acid for reducing blood loss following vaginal delivery: a double-blind randomized controlled trial

**DOI:** 10.1186/s12884-022-04462-z

**Published:** 2022-03-03

**Authors:** Francis Nwabueze Igboke, Vitus Okwuchukwu Obi, Benedict Ikechukwu Dimejesi, Lucky Osaheni Lawani

**Affiliations:** 1 Alex-Ekwueme Federal University Teaching Hospital, Abakaliki, Nigeria; 2 Institute of Health Policy, Management and Evaluation, University of Toronto, Toronto, ON M5T 3M6 Canada

**Keywords:** Tranexamic acid, Postpartum haemorrhage, Blood loss, Vaginal delivery, Prevention

## Abstract

**Background:**

Postpartum haemorrhage (PPH) is a major cause of maternal morbidity and mortality worldwide. Tranexamic acid (TXA) is a useful drug for prevention of PPH and merits evaluation in Nigeria, where PPH is the leading cause of maternal death (25%) and severe maternal morbidity. This study evaluates the efficacy of TXA in reducing blood loss following vaginal delivery.

**Methods:**

This was a double-blind randomized placebo-controlled study on the efficacy and safety of intravenous TXA in reducing blood loss in women undergoing vaginal delivery in a tertiary hospital. Data analysis was conducted with IBM SPSS software (version 20, Chicago II, USA). *P*-value < 0.05 was considered statistically significant.

**Results:**

The mean estimated blood loss was lower in TXA compared with the placebo group. (174.87 ± 119.83 ml versus 341.07 ± 67.97 ml respectively; *P* < 0.0001). PPH (blood loss > 500 ml) was 5.13% in the study arm compared to the control arm 7.14%- risk ratio (RR) 0.71; 95% CI: 0.38–1.79, *p* = 0.5956]. Additional uterotonics was required more in the control group compared to the treatment group 14(16.67%) versus 3(3.85%), *p*-value= 0.007. There were no major complications noticed in the treatment group.

**Conclusion:**

This study demonstrated that intravenous administration of TXA reduced blood loss following vaginal delivery. It also reduced the need for additional uterotonics. However, blood loss greater than 500 was not significantly reduced.

**Trial registration:**

This trial was registered retrospectively.

Pan African Clinical Trial Registry: PACTR202010828881019 on 12/10/2020.

## Background

Globally, about 500,000 women die yearly from complications of pregnancy and childbirth [1]. Majority of these deaths occur in the immediate postpartum period and in most cases are due to postpartum haemorrhage (PPH) [2, 3]. PPH is the commonest cause of maternal death, with the highest incidence in low-middle-income countries (LMIC) [1, 4]. Moreover, postpartum haemorrhage (PPH) is the main cause of severe maternal morbidity (SMM), accounting for 47.6% of the cases of SMM [5, 6]. PPH is the excessive bleeding per vaginam after the delivery of the baby and up to six weeks postpartum [1]. It can either be primary or secondary [6, 7]. Primary PPH is the loss of > 500mls of blood within the first 24 h of delivery or loss of any amount that is enough to cause haemodynamic instability [7, 8]. Primary PPH complicates approximately 3% of vaginal deliveries [9].

In many cases of PPH, the true blood loss is often underestimated due to problems with visual blood estimation [10–12]. The risk of severe maternal morbidity and death from PPH depends on the amount and rate of blood loss, as well as the clinical state of the woman [10], such that blood loss of as little as 200 ml could be life-threatening in woman with severe anaemia or cardiac disease [13]. The risk factors for PPH includes previous PPH, primiparity, prolonged or augmented labour, multiple pregnancy, previous caesarean delivery, polyhydramnios, and macrosomia [14]. Nevertheless, most women with PPH have low-risk pregnancies and no identifiable risk factors. It is therefore important to prevent PPH in all women [15].

The interval between delivery and placental expulsion is a critical window for the prevention of PPH [16]. Oxytocics and anti-fibrinolytic like Tranexamic acid (TXA) are effective in prevention and treatment of PPH [17–19]. Active management of the third stage of labour (AMTSL) as one of the strategies for the prevention of PPH. It comprises of the administration of uterotonic agents after delivery of the baby, delayed cord clamping and cutting and controlled cord traction (CCT) [20–23]. In addition to this enhancement of mechanical haemostasis, a supportive biochemical haemostatic effect might also be expected from the complementary use of prohaemostatic drugs such as TXA in the prevention of PPH [24].

Tranexamic acid is a potent antifibrinolytic agent that exerts its effect by blocking lysine binding sites on plasminogen molecules preventing the breakdown of blood clot (fibrinolysis) and resulting in hemostasis [24]. Previous trials have shown that TXA in planned surgery reduces the risk of blood transfusion, mean transfused volume, and need for re-operation due to bleeding without safety concerns [25, 26]. Furthermore, considerable decrease in mean menstrual blood loss have been reported in women with menorrhagia treated with TXA, in contrast to control or placebo-treated women [27–29]. A randomized controlled trial (RCT) involving studies in high-income countries demonstrated the efficacy of TXA in prevention of PPH [30]. However, the authors reports that small sample size was a limitation of the study. Therefore, there is need for adequately powered studies with larger representative sample size, especially in a low resource setting like Nigeria. The present study aims to evaluate the efficacy and safety of TXA in prevention of PPH and associated SMM after vaginal deliveries in a Nigerian setting.

## Methods

### Study design

This was a double-blind randomized placebo-controlled study on the efficacy and safety of intravenous TXA in reducing blood loss in women undergoing vaginal delivery at a tertiary hospital in southeast Nigeria and who met the inclusion criteria after obtaining an informed consent. The study was conducted from June 2018 to December 2019. The study was approved by the Human Research and Ethics Committee (HREC) of Alex-Ekwueme Federal University Teaching Hospital, Abakaliki (FETHA/REC/VOL1/2017/541).

This study was registered with Pan African Clinical Trial Registry: PACTR202010828881019.

### Study setting

The study was conducted in the department of Obstetrics and Gynaecology, Alex-Ekwueme Federal University Teaching Hospital, Abakaliki (AEFUTHA), Nigeria. The department manages both low and high-risk pregnant women using standardized protocols.

### Participants

The participants for this study were from the population of women within the reproductive age group undergoing vaginal delivery at the Alex-Ekwueme Federal University Teaching Hospital, Abakaliki who met the inclusion criteria after obtaining an informed consent.

The inclusion criteria include spontaneous labour in booked patients, planned vaginal delivery, term pregnancy, singleton pregnancy and cephalic presentation, parturient who have no contraindications to the use of tranexamic acid and informed consent form signed. Women with prior history of thromboembolism, autoimmune diseases, sickle cell disease, bleeding disorders, renal disease, liver pathology, known cardiovascular disease, multiple pregnancy, intrauterine fetal death, previous uterine surgeries, patients with chronic hypertension, preeclampsia, eclampsia, HELLP syndrome, antepartum haemorrhage, ruptured uterus, varicose veins at increased risk of deep vein thrombosis, history of epilepsy/seizures and those that had episiotomy were excluded.

### Sample size

The minimum sample size was determined using the formula for comparison between two groups when the end point is a quantitative data [31].

$$\begin{aligned}\mathrm S\mathrm a\mathrm m\mathrm p\mathrm l\mathrm e\;\mathrm s\mathrm i\mathrm z\mathrm e={2\mathrm S\mathrm D}^2\;\left(Z_{\alpha/2}\;+\;Z_\beta\right)^2/\text{d}^2\end{aligned}$$

Where:

SD: Standard deviation of blood loss for treatment group was 32.4; derived from a pilot study conducted on use of tranexamic acid in prevention of postpartum haemorrhage in a similar population of women who had caesarean section at the study centre (AEFUTHA).

Z_*α/2*_: 1.96 (from Z table) at type 1 error of 5%

Z_*β*_: To increase accuracy of the study, 90% power was used = 1.282 (from Z table).

d: Standardized effect size (difference in mean value between 2 groups- Sentilhes et. al) = 236.9 (placebo arm)—220.3 (treatment arm) [6]= 16.6.

Sample size per group = 2 (32.4)^2^ x (1.96 + 1.282)^2^ / (16.6)^2^=80

Ten percent (10%) of the minimum sample size per group (10/100 × 80/1≈8) was added to correct for any attrition hence the final sample size was 88 for each arm.

### Randomization and concealment

The participants were randomized by means of a computer-generated random number using the software Research Randomizer®. Eighty-eight (88) numbers were randomly generated from a pool of one hundred and seventy-six (1–176) and these numbers were assigned to group A (tranexamic acid group), while the remaining eighty-eight were automatically assigned to group B (the placebo group).

Group A received 1 g tranexamic acid (Exacyl®; Sanofi Aventis Paris France) slowly (over 30–60 s) intravenously, within 2 min after birth and prophylactic oxytocin administration once the cord had been clamped.

Group B received 10mls of water for injection (Biofem®; Juhel Anambra Nigeria) slowly (over 30–60 s), within 2 min after birth and prophylactic oxytocin administration, once the cord had been clamped. These drugs were sourced from their drug representatives.

Concealment was done in sequentially numbered opaque sealed envelopes (SNOSE) [32]. These numbers (1–176) were inscribed on brown envelopes and a piece of paper with the inscription ‘tranexamic acid’, or ‘placebo’ was placed with the respective drug or placebo accordingly inside these envelopes and sealed. The randomization was done by a statistician and an obstetrician, while the concealment was done by a hospital pharmacist without revealing the results to the researchers. All the envelopes were kept in a locker that was made accessible to all the members of the research team.

Participants that met the inclusion criteria having signed the informed consent form were given sequential study number and the corresponding numbered opaque sealed envelope was allocated to the patient.

### Study procedure

Women were selected for vaginal delivery in the facility and admitted into the labour ward. They were counselled on the study and those who signed the informed consent form were recruited. The antenatal card was retrieved, and highlights reviewed. History was taken and clinical examination was done to confirm the stage of labour while ancillary investigations; haematocrit, haemoglobin and urinalysis were done, and patients were transferred into the labour ward in active phase of labour. The labour was managed actively with the partograph, and augmentation was done as indicated.

The researchers or any of the research assistants took the allotted sealed envelope to the labour ward and handed same over to the labour ward officer who administered the drug or the placebo over 30 to 60 s within 2 min of delivery of the baby. The envelope with its used content (resealed) was returned to the investigators who kept all the used envelope/packs in a separate locker until the end of the study when un-blinding was done.

AMTSL was carried out for all recruited patients according to departmental protocol (cord clamping, use of oxytocin and controlled cord traction) [30]. Other oxytocics and surgical interventions required to control excessive bleeding were given or done and patients who needed blood transfusion received same.

These interventions were noted. Immediately after the delivery of the baby, when all the liquor were drained, a blood drape (an improvised BRASS-V, a disposable conical, graduated plastic collection bag) was inserted under the patient [30, 33]. The blood collector drapes were applied in between delivery of the baby to delivery of the placenta/repaired of genital laceration or episiotomy and removed after all events associated with third stage of labour were completed i.e. after the woman is cleaned up and a perineal pad put in place. The blood collected in the blood drape was transferred into a transparent plastic measuring cylinder with a capacity of 500 ml, corrected to 2 ml and manufactured by Measure Masters®. The blood in the measuring cylinder was read off and documented by the researcher or the assistants. Then, the patient was given pre-weighed pads, which were re-weighed 2 h post-partum [30]. The assessment of blood loss through weighing of sanitary pads within 2 h of delivery was based on the concept of 4^th^ stage of labour [34], where uterine contractility and hemodynamic status (vital signs) are monitored for 1–2 h postpartum while the woman is still in the labour and delivery unit. This is the period when the woman is at the greatest risk of primary PPH and therefore require close monitoring and assessment. For uniformity, the regular labour ward sanitary pad with negligible dry weight was used. EBSA-20 electronic weighing scale, which operates at room temperature with readability of 20 kg and 5 g for maximum and minimum weights respectively, manufactured by the Zhongshan Jimli Electronic Weighing Equipment Co. Ltd was used.

The side effects of the drug were noted. The patients were transferred to the post-natal ward for further observation. The patients’ post-delivery pulse rate and blood pressure were noted and recorded [32].

### Blood loss estimation

The estimated blood loss ascertained by measuring the blood collected in the drape and complimented by measuring the weights of the sanitary pads before and after 2 h of delivery. Immediate post-partum blood loss was calculated thus [32].

Total blood loss (ml) = Blood in the measuring cylinder (ml) + [ Pad weight after 2 h (gm)-Pad weight prior to use (gm)]- converted to ml; taking that 1 g difference in pad weight equals 1 ml of blood [33].

Blood loss greater than > 500 ml was be regarded as excessive bleeding (postpartum haemorrhage).

### Follow up

Patients were expected to stay for 48 h on admission in the hospital before discharge except otherwise indicated. The duration of stay was dependent on the patient’s clinical state. The participants were followed up until discharge from the facility. They were instructed to present to the hospital or reach the researchers or any of the research assistants by phone if they have any unforeseen adverse reaction which was to be reported.

### Outcome measures

Primary outcome measure was estimated blood loss following vaginal delivery (total blood loss following vaginal delivery = estimated blood from cylinder + difference in the weight of pad) [32]. Secondary Outcome Measures include primary PPH following vaginal delivery defined as blood loss > 500 ml, need for additional uterotonics to control bleeding, need for blood transfusion (volume and amount) after vaginal delivery, mild maternal side effects (nausea, vomiting, headache, skin rash), major maternal side effects (thromboembolism, maternal death) [35].

### Statistical analysis

Data was collated, tabulated, and statistically analyzed with the Statistical Package for Social Science (IBM SPSS) software (version 20, Chicago II, USA). Continuous variables as the maternal vital signs were presented as means and standard deviations (Mean ± 2SD), while categorical variables like minor and major side effects were presented as numbers and percentages. Chi-square test (X^2^) was used for comparison between groups for qualitative variables while t-test was used for comparison between groups for quantitative variables. A difference with a *p*-value < 0.05 was considered statistically significant.

## Results

**Fig. 1:**
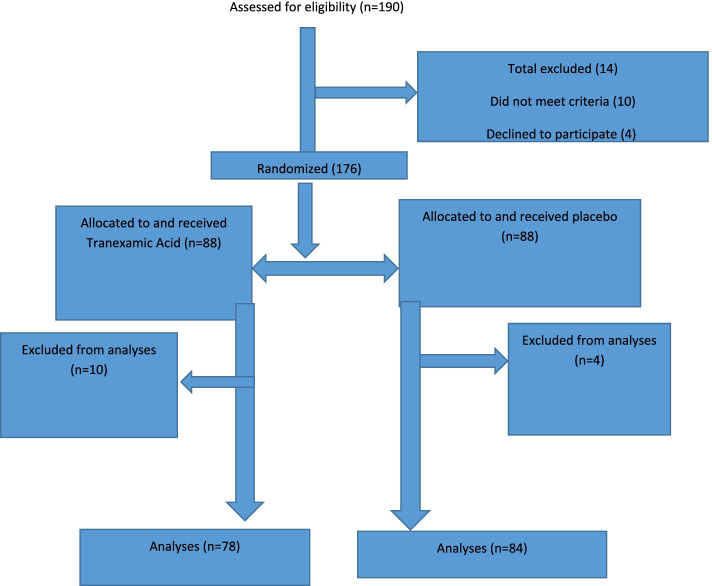
Flow of patients through the study

Over the study period of 6 months, 190 patients were assessed for randomization into the study; 14 were excluded while 176 were allocated to receive either TXA or placebo. Only 78 in the study group and 84 in the control (placebo) group were available for final analysis (Fig. 1).

The demographic characteristics of the participants- maternal age, parity, gestational age, and height as well as the fetal birth weight are presented in Table 1. The participants were matched to ensure the baseline maternal characteristics between matched pairs were similar.

**Table 1:** Demographic characteristics of the patients

Variables	Study group, *N* = 78 (mean ± SD)	Placebo group, *N* = 84 (mean ± SD)
Maternal age (years)	27.95 ± 5.10	29.95 ± 3.60
Gestational age (weeks)	39.01 ± 1.38	39.01 ± 1.33
Height (metres)	1.59 ± 0.05	1.59 ± 0.06
Weight (kg)	78.68 ± 9.90	80.75 ± 12.01
Fetal birth weight (kg)	3.26 ± 0.39	3.25 ± 0.45
Parity		
0	17	20
1–4	61	64

The mean systolic blood pressure (BP) at presentation, 1 h and 2 h post- delivery were not significantly different. Also, the diastolic BP at presentation, 1 h and 2 h postpartum as well as the pulse rates between the two groups were not different at these times (Table 2).

**Table 2:** Maternal vital signs at different times before and after delivery

Maternal vital signs	Study group, *N* = 78	Placebo, *N* = 84
**On Admission**		
Pulse rate (beats per minute)	87.27 ± 5.96	86.33 ± 5.47
Systolic BP (mmHg)	112.76 ± 9.76	113.40 ± 10.42
Diastolic BP (mmHg)	77.26 ± 8.49	79.05 ± 7.86
**1 h after delivery**		
Pulse rate (beats per minute)	86.18 ± 6.13	87.07 ± 5.08
Systolic BP (mmHg)	112.63 ± 8.60	112.83 ± 9.72
Diastolic BP (mmHg)	75.64 ± 7.83	76.21 ± 7.14
**2 h after delivery**		
Pulse rate (beats per minute)	85.58 ± 5.73	84.74 ± 4.54
Systolic BP (mmHg)	111.513 ± 7.82	111.31 ± 8.14
Diastolic BP (mmHg)	74.52 ± 6.74	74.14 ± 6.64

The mean duration of application of the blood collection drapes were 28.2 ± 6.4 min and 29.4 ± 5.4 min for the treatment and placebo groups respectively. The mean estimated blood loss was significantly lower in the TXA group compared with the placebo group (174.87 ± 119.83 ml versus 341.07 ± 67.97 ml respectively; *P* < 0.0001), mean difference of 166.2 ml (48.7%). There was no significant statistical difference between the mean haematocrit of the treatment group versus the control group at presentation (33.99 ± 3.00 versus 34.01 ± 2.92, *p* = 0.9658), however, the mean haematocrit between the two groups 48 h postpartum was statistically different (32.54 ± 3.36 versus 31.33 ± 2.88, *p* = 0.0147). The mean change in haematocrit between the groups 48 h after delivery was also significantly different (3.14 ± 0.94 versus 4.11 ± 1.1, *p* = 0.0018). The haemoglobin concentration at presentation between the two groups was not significantly different (11.66 ± 1.00 versus 11.84 ± 0.90 for treatment and control groups respectively, *p* = 0.2297). However, the haemoglobin concentration 48 h after delivery was significantly different (11.14 ± 1.07 versus 10.45 ± 0.96 for treatment and control groups respectively, *p* =  < 0.0001). There was no significant difference in the platelets and the risk of bleeding between both groups at presentation (Table 3).

**Table 3:** Pre delivery and post-delivery Haemoglobin/Haematocrit levels in the study and in the control group

Variables	Study group, *N* = 78 (mean ± SD)	Placebo group, *N* = 84 (Mean ± SD)	*P*-value
Blood loss at delivery (ml)	174.87 ± 119.83	341.07 ± 67.97	< 0.0001
**Maternal Haematocrit (%)**			
Pre-delivery	33.99 ± 3.0	34.01 ± 2.92	0.9658
48 Hours Postpartum	32.54 ± 3.36	31.33 ± 2.88	0.0147
Mean Change in Haematocrit	3.14 ± 0.94	4.11 ± 1.1	0.0018
**Maternal Haemoglobin (g/dl)**			
Pre-delivery	11.66 ± 1.00	11.84 ± 0.90	0.2297
48 h postpartum	11.14 ± 1.07	10.45 ± 0.96	< 0.0001
Difference in Haemoglobin after 48 h	0.94 ± 0.43	1.21 ± 0.63	0.0019
Platelets	198.7 ± 36.5	203.1 ± 43.7	0.4895
Clothing Time	4.5 ± 0.9	4.7 ± 1.1	0.2092

Blood loss > 500 ml was not significantly higher in the study group compared to the control group (Risk ratio (RR) = 0.71, CI [0.38–1.79], *p* = 0.5956 (Table 4).

**Table 4:** Pre-delivery and post-delivery variables in the study and control groups

Variable	Study group *N* = 78, n (%)	Placebo group *N* = 84, n (%)	RR (95%CI)	*P*-value
Blood loss > 500 (ml)	4(5.13%)	6(7.14%)	0.71(0.38–1.79)	0.5956
**Additional interventions**				
Blood transfusion	1(1.28%)	3(3.57%)	0.25(0.09–2.82)	0.3496
Uterotonics	3(3.85%)	14(16.67%)	0.24(0.12–0.96)	0.007
**Patients with side effects**				
Minor side effects: Diarrhoea	1(1.15%)	0(0%)		

There was no significant difference in the blood transfusion received by both groups, RR = 0.25 (0.09–2.82), *p* = 0.3496. Additional uterotonics was required more in the control group compared to the treatment group, RR = 0.24(0.12–0.96), *p* = 0.007 (Table 4).

There were no major complications noticed in the treatment group. However, diarrhoea was noticed only in one patient in that group (Table 4).

## Discussion

In this study, administration of 1 g intravenous TXA prior to placenta delivery after delivery of the baby was associated with a 48.7% (166.2 ml) reduction in blood loss at vaginal delivery compared to placebo. This reduction was higher than the 25.3% (*p* < 0.001) reported by Gungorduk and colleagues [35], 22.3% (*p* < 0.01) and 21.3% (*p* < 0.03) reported by Yang and co-workers [36] and Mirghafourvand and colleagues [30] respectively. These difference in blood loss may be as a result of the different time intervals for administration of TXA. In the present study, TXA was administered within 2 min of delivery of the baby. Gungorduk and colleagues [35] documented TXA administration of 5 min while Mirghafourvand and colleagues documented 10 min after delivery of the anterior shoulder [30]. The WOMAN trial had recommended early administration of tranexamic acid in the management of severe bleeding following delivery. It may also be due to the time interval of assessing the blood loss and the different methods used in the estimation of blood loss for the various studies.

While some used graduated bags [28, 30], others calculated the mean blood loss volume by measuring sheets of pads from the end of delivery to 2 h after birth [32]. This study also incorporated the change in haematocrit after 48 h of delivery.

Blood loss of > 500 ml was not significantly reduced in the study group when compared to the control group. This finding is similar to the finding made by Mirghafourvand and colleagues [30] (*p* = 0.14) but contradicted the finding noted by Gungorduk and co- workers [35] in which the reduction of blood > 500 ml was statistically significant (*p* < 0.01). This could have been because Gungorduk et al. incorporated high risk parturient who were likely to bleed more into their study which was not the case in this study [35].This study showed that prophylactic administration of TXA after the delivery of the baby and before the delivery of the placenta reduced the need for additional uterotonics following vaginal delivery. This is consistent with the findings in most studies that compared the efficacy of TXA to placebo in reducing blood loss after vaginal delivery [28, 30, 32, 35, 37]. Although a patient needed blood transfusion in the treatment group due to primary PPH following retained placenta, but this could also have happened to either group. A similar finding was documented by Gungorduk et al. [35] However, Sentilhes et al [28] and Roy et al [32] observed that the use of TXA reduced the need for blood transfusion.

There was a statistically significant difference in the mean haematocrit and haemoglobin 48 h after delivery between the two groups. This was consistent in the findings of other studies [27, 29, 35–37]. There was no significant difference in the vital signs of the patients on admission, one hour and two hours postpartum between the placebo and the study groups. However, the use of TXA was associated with a small increase in the risk of minor side effects (majorly diarrhoea) in this study but this was not statistically significant. Other studies also reported minor maternal gastrointestinal side effects and were not documented as significant [29, 34, 35]. There were no major maternal side effects or maternal death recorded in the present study. This was also the finding by similar studies [28, 30, 36, 37] and suggests that TXA did not have any adverse maternal outcome.

The limitations are that the study was a single centre randomized controlled study, it did not evaluate the efficacy of intravenous TXA in high-risk patients. Liquor and lochia contamination of the measured blood may not have been completely avoided. In addition, the assessment of blood loss was limited to within 2 h of delivery.

The main strength of this study includes the internal validity and reliability of the findings, as well as external validity which makes it generalizable.

In summary, this study demonstrated that intravenous administration of TXA acid following the delivery of the baby and before delivery of the placenta reduced blood loss following vaginal delivery. It reduced the need for additional uterotonics to control blood loss. However, the incidence of primary postpartum haemorrhage and the need for blood transfusion was not significantly reduced between the two groups. Minor side effect  like diarrhoea was noted but this was not statistically significant between the groups. There were no major maternal side effects, and no maternal death was recorded. Given the findings of this study, we reject the the null hypothesis. We therefore conclude that this result lays credence to the fact that intravenous TXA used to prevent primary PPH is safe and effectively reduced blood loss following vaginal delivery without increasing maternal risks and should be made available for women selected for vaginal delivery as no woman is immune to postpartum haemorrhage. Furthermore, that there is need to evaluate these findings on a larger scale for prophylactic purposes. Finally, further research is needed to evaluate the efficacy and safety of TXA in women at risk of excessive blood loss and anaemic patients in our setting and to evaluate blood loss from delivery up to 24 h after delivery.

## Data Availability

The data used or analyzed during the current study are included within the article. The datasets are not publicly available due to the hospital policy and personal privacy. However, the datasets are available from the corresponding author on reasonable request.

## Abbreviations

TXA: Tranexamic acid
PPH: Postpartum haemorrhage
AEFUTHA: Alex-Ekwueme Federal University Teaching Hospital Abakaliki
RCT: Randomized controlled trial
AMTSL: Active management of third stage of labour
HREC: Human research and ethics committee
CI: Confidence Interval
